# Diagnostic Pitfalls of Prosthetic Valve Endocarditis: From Sacroiliitis to Coronary Septic Embolization

**DOI:** 10.3390/diagnostics15202620

**Published:** 2025-10-17

**Authors:** Camelia Bianca Rus, Corina Cinezan

**Affiliations:** 1 Department of Medical Disciplines, Faculty of Medicine and Pharmacy, University of Oradea, 410073 Oradea, Romania; 2 Clinical County Emergency Hospital Bihor, 410169 Oradea, Romania; 3 Doctoral School of Biological and Biomedical Sciences, University of Oradea, 410087 Oradea, Romania

**Keywords:** prosthetic valve endocarditis, transcatheter aortic valve implantation, myocardial infarction, septic embolization, percutaneous coronary intervention, culture-negative endocarditis, high-risk patients

## Abstract

Background: Transcatheter aortic valve implantation (TAVI) is an established treatment for severe aortic stenosis in elderly and high-risk patients. However, prosthetic valve endocarditis (PVE) remains a rare but devastating complication. Its diagnosis is often delayed due to atypical clinical manifestations and the frequent occurrence of culture-negative endocarditis. Case Presentation: We report the case of a 68-year-old woman with a prior TAVI who presented with sacroiliitis, initially interpreted as a localized musculoskeletal infection. Subsequent evaluation revealed infective endocarditis involving the prosthetic aortic valve and the native mitral valve. Blood cultures remained negative, most likely due to prior antibiotic therapy, which complicated timely diagnosis. During hospitalization, the patient developed acute ST-segment elevation myocardial infarction (STEMI), caused by coronary septic embolization. Discussion: Distinguishing septic emboli from thrombotic occlusion in the setting of STEMI complicating endocarditis is extremely challenging but essential, as therapeutic approaches diverge. While percutaneous coronary intervention is the standard treatment for thrombotic occlusion, it carries major risks of septic embolization, including stent infection, mycotic aneurysm, and uncontrolled sepsis. Conclusions: This case highlights the need for high clinical suspicion of PVE in atypical presentations, the diagnostic challenges of culture-negative endocarditis, and the therapeutic dilemmas posed by acute coronary complications without clear guideline-based solutions.

## 1. Introduction

Aortic stenosis (AS) is the most prevalent valvular disease in developed countries, primarily caused by progressive age-related calcification, but also by congenital bicuspid valve or rheumatic disease [1,2]. Severe symptomatic AS carries a poor prognosis without intervention, and aortic valve replacement remains the only definitive therapy. While surgical aortic valve replacement (SAVR) has long been the standard of care, TAVI has become the preferred option in elderly and high-risk patients, owing to its less invasive nature and expanding indications [3,4,5].

Despite these advances, both SAVR and TAVI are associated with specific complications. Among them, PVE remains a serious and life-threatening condition, with an incidence of 0.3–2.3% per patient-year after valve replacement [6,7]. TAVI-related PVE is increasingly recognized and carries distinctive risk factors, such as advanced age, vascular access, paravalvular leak, and prosthesis design [8].

The clinical recognition of PVE is often demanding, especially in late endocarditis, because clinical presentation is often atypical, diagnostic confirmation is difficult, and management is particularly challenging. Such patients may present with atypical symptoms and signs, such as musculoskeletal involvement including sacroiliitis, rather than classic features of endocarditis. In this context, delayed diagnosis is common and can be fatal, especially in fragile post-TAVI patients. Furthermore, negative blood cultures—frequently resulting from prior antibiotic exposure for unrecognized or misinterpreted symptoms—represent an additional diagnostic challenge and may significantly delay appropriate treatment [5].

A particularly severe manifestation of PVE is ST-elevation myocardial infarction (STEMI), most often due to coronary embolization. In this scenario, differentiating between thrombotic occlusion and septic embolization is crucial, as management strategies differ fundamentally. While urgent revascularization is standard in thrombotic STEMI, coronary intervention in septic embolization carries high risks, including infection dissemination, mycotic aneurysm, and poor outcomes [6,7].

This case highlights the importance of maintaining high clinical suspicion for PVE in patients with atypical presentations and negative blood cultures and emphasizes the need for multidisciplinary decision-making when STEMI complicates prosthetic valve infection.

## 2. Methods

Clinical data were obtained through direct patient assessment, review of medical records, and analysis of laboratory and imaging results. The diagnostic process and therapeutic interventions were carried out according to current clinical guidelines and institutional protocols. Informed consent for the publication of this case report was obtained from the patient.

## 3. Case Report

We report the case of a diabetic 68-year-old female who was admitted to the Emergency Department Unit at the Clinical County Emergency Hospital Bihor, complaining of the following symptoms: fatigue, dyspnea, and profuse sweating. Her past medical history included TAVI three years earlier with good initial recovery.

### 3.1. Medical History

Two months prior to this admission, she had been hospitalized in the Infectious Disease Department with symptoms like fatigue, gait disorder, and lumbago. At that time the elevated levels of C-reactive protein (CRP): 14.2 mg/L in the blood in addition to her clinical presentation and Magnetic Resonance Imaging (MRI) led to the diagnosis of sacroiliitis and spondylodiscitis, for which she received intravenous antibiotics (clindamycin and gentamicin). Importantly, these antibiotics were administered before blood cultures were obtained, which subsequently contributed to culture-negative infective endocarditis (IE) and delayed recognition of the true underlying disease.

### 3.2. Clinical Case

At the time of admission, the patient was hemodynamically stable: blood pressure of 135/76 mmHg, respiratory rate 33/min, pulse 99 bpm, SaO_2_ 88% on room air. Multiple blood tests and imaging investigations were performed in order to make a correct diagnosis. The laboratory exam is shown in Table 1. Blood tests were significant for the following: hemoglobin 11 g/dL, C-reactive protein 28 mg/L, Alanine Transaminase (ALAT) 191 U/L, Aspartate Transaminase (ASAT) 241 U/L, Gamma-Glutamyl Transferase (GGT) 300 U/L, creatinine 1.53 mg/dL, and Serum Urea 40 mg/dL. Computed tomography (CT) of the lungs revealed the presence of bilateral, moderate pleural effusions and CT pulmonary angiogram (CTPA) revealed a pulmonary embolism (PE) in the left tributary lung artery.

**Figure 1:**
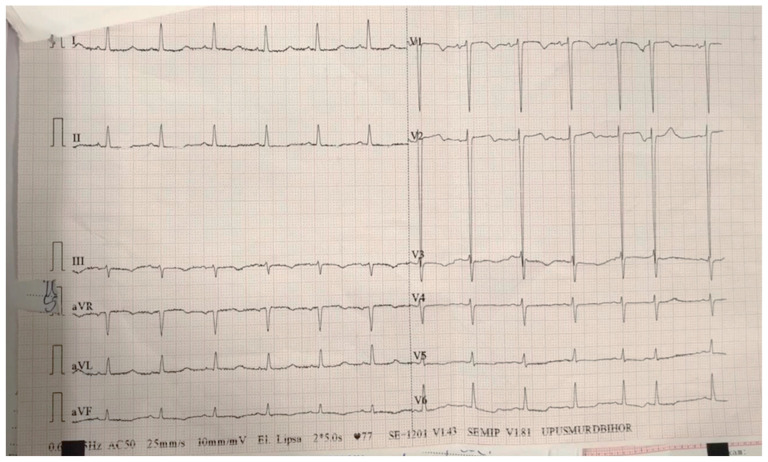
ECG on admission—sinus rhythm, heart rate 77 bpm, normal axis, deep S waves in leads V1–V2 suggestive for left ventricle hypertrophy.

Electrocardiogram (ECG) showed normal sinus rhythm, with left ventricle hypertrophy (Figure 1).

**Figure 2:**
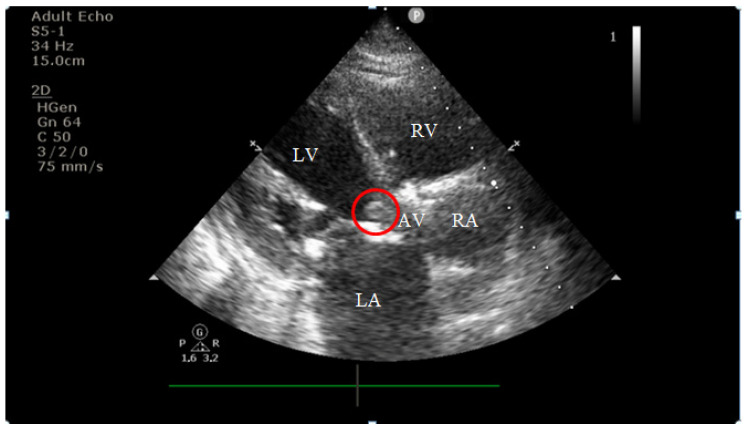
Echocardiography on admission showing a hyperechogenic, mobile, irregular mass attached to the aortic prosthetic valve; LV—left ventricle; RV—right ventricle; LA—left atrium; RA—right atrium; AV—aortic valve; red circle—hyperechogenic, mobile, irregular mass attached to the aortic prosthetic valve, measuring 8 × 16 mm.

**Figure 3:**
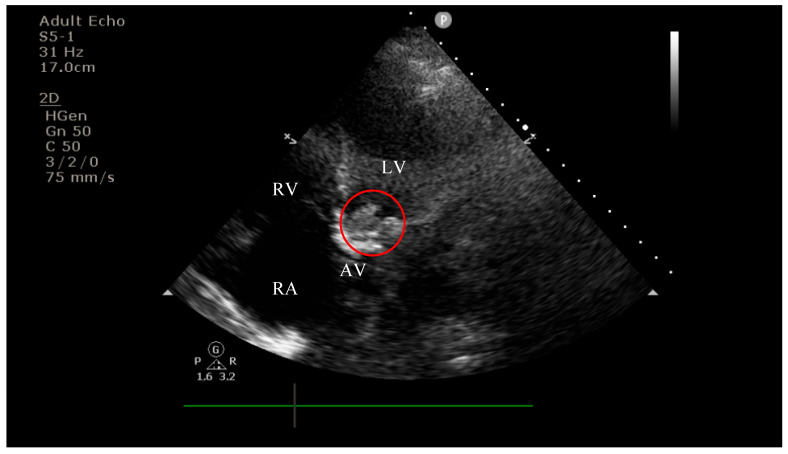
Apical four-chamber view showing a hyperechogenic, mobile, irregular mass attached to the aortic prosthetic valve; LV—left ventricle; RV—right ventricle; RA—right atrium; AV—aortic valve; red circle—hyperechogenic, mobile, irregular mass attached to the aortic prosthetic valve.

**Figure 4:**
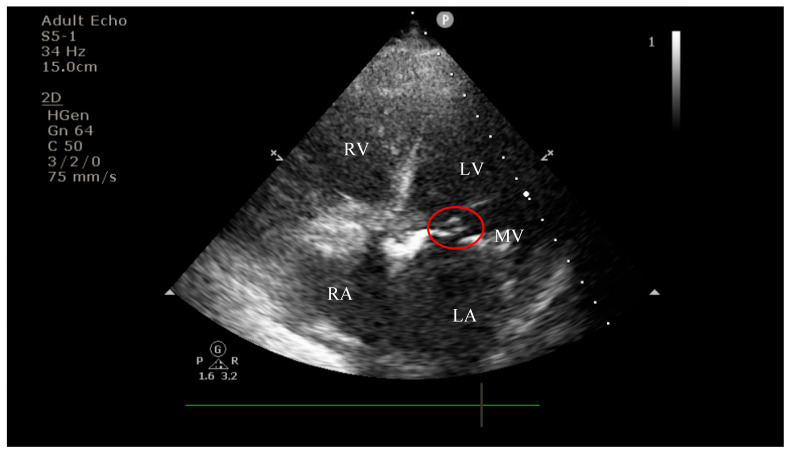
Apical four-chamber view showing a hyperechogenic, mobile, irregular mass attached to the mitral valve; LV—left ventricle; RV—right ventricle; LA—left atrium; RA—right atrium; MV—mitral valve; red circle—hyperechogenic, mobile, irregular mass attached to the mitral valve, measuring 8.5 × 8.2 mm.

**Figure 5:**
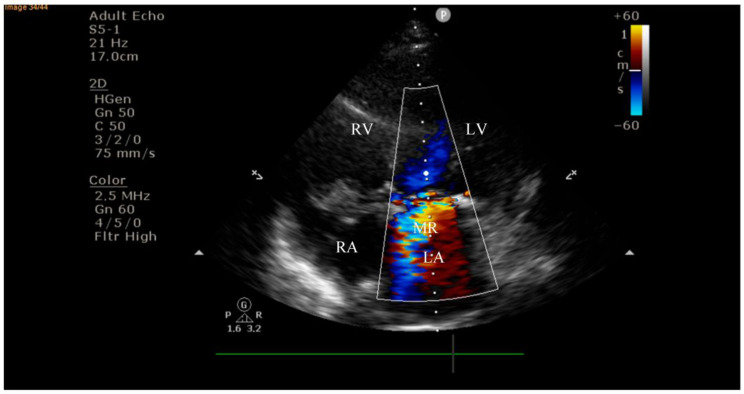
Apical four-chamber view on admission demonstrating severe mitral regurgitation with a large eccentric regurgitant jet directed into the left atrium; LV—left ventricle; RV—right ventricle; LA—left atrium; RA—right atrium; MR—mitral regurgitation jet.

**Figure 6:**
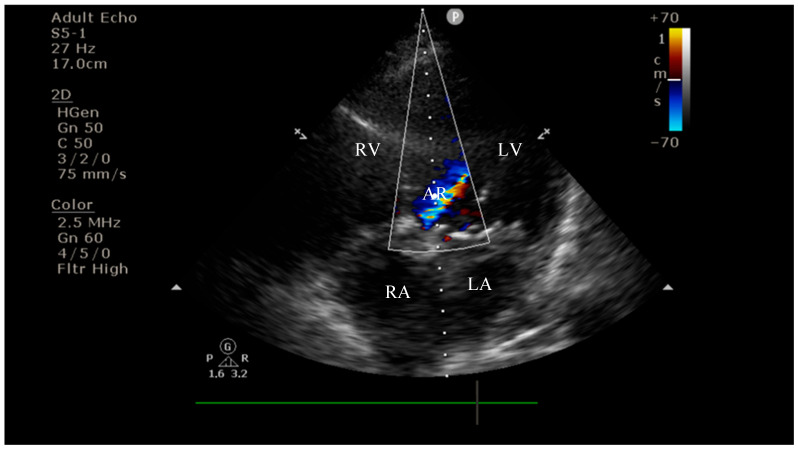
Apical four-chamber view on admission demonstrating moderate aortic regurgitation; LV—left ventricle; RV—right ventricle; LA—left atrium; RA—right atrium; AR—aortic regurgitation jet.

On current admission, transthoracic echocardiography revealed vegetations on both the prosthetic aortic valve (8 × 16 mm)—Figure 2 and Figure 3 and the anterior mitral leaflet (8.5 × 8.2 mm)—Figure 4, with associated severe mitral regurgitation—Figure 5 and moderate aortic regurgitation—Figure 6. She was diagnosed with prosthetic and native valve IE, complicated by pulmonary embolism. The patient was admitted to the Intensive Cardiac Care Unit for close monitoring and advanced support.

**Figure 7:**
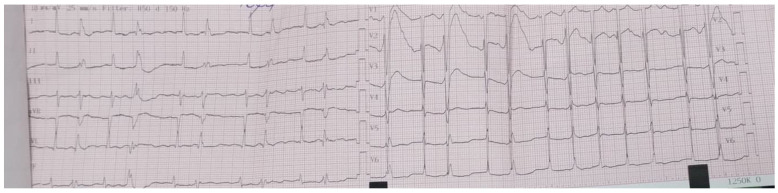
ECG on the third day of hospitalization—atrial fibrillation, heart rate 150 bpm, normal axis, isolated ventricular extrasystoles.

After admission, multiple blood cultures (at least two) were drawn to identify the causative organism, and Empiric Therapy with broad-spectrum antibiotics was started before targeting the likely organisms, which were staphylococci, streptococci, and enterococci, as the ESC 2023 Guideline [9] recommends. The initial antibiotic therapy included the following: Ceftriaxone 2 g twice a day, Gentamicin 80 mg twice a day, and Ampicillin 2 g four times a day (renal adjusted dose). Apart from the empiric antibiotic therapy, treatment for heart failure and pulmonary embolism was initiated too. Oxygen therapy was administered to address hypoxemia as SpO_2_ levels persistently stayed under 90%. Despite empiric guideline-based antibiotic therapy, her clinical course was rapidly progressive. The patient developed nausea, vomiting, and tachycardia associated with dyspnea and hemodynamic instability requiring inotropic support and slowly progressing to cardiogenic shock. ECG revealed atrial fibrillation with high heart rate (Figure 7) so we initiated pharmacological cardioversion using Amiodarone.

We repeated blood tests at 48 h in order to monitor possible changes. The results are shown in Table 2. They show lower transaminase levels and normal creatinine and serum urea but higher C-reactive protein levels of 48 mg/L.

**Figure 8:**
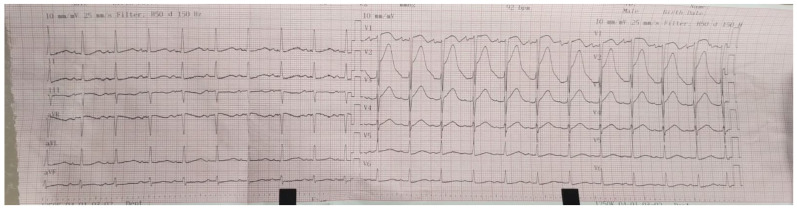
ECG on the sixth day of hospitalization—sinus rhythm, heart rate 100 bpm, normal axis, ST-segment elevation in leads V1–V3.

On the sixth day of hospitalization, the patient started to complain of very intense anterior chest pain, nausea and sweats. An ECG was performed (Figure 8). It showed ST-segment elevation in leads V1–V3.

An emergent coronary angiogram was performed; it showed near-total occlusion of the left anterior descending artery, presumably due to a septic embolus. In the absence of clear angiographic criteria to differentiate septic embolization from thrombotic occlusion, a drug-eluting stent was implanted after antiplatelet loading. Post-intervention, her condition deteriorated to refractory cardiogenic shock, and she died 15 h later.

## 4. Discussion

This case highlights numerous challenges in diagnosing and managing IE in patients with TAVI. IE after TAVI is relatively uncommon, but when it occurs, leads to a high mortality rate, often associated with an atypical presentation and difficulty in diagnosing [10,11]. Our patient developed IE on a biological aortic valve implanted via TAVI and on a native mitral valve, eventually complicated by severe mitral regurgitation, acute myocardial infarction and death, emphasizing the severity of this condition. There are several factors that can make this case highly educational.

### 4.1. Atypical Presentation and Diagnostic Delay

Our patient had multiple encounters with the health care system prior to the diagnosis, including three presentations to the Infectious Disease Department where she was diagnosticated with sacroiliitis and she received appropriate treatment for it. It is likely that the patient’s first manifestation was sacroiliitis and spondylodiscitis, an under-recognized early feature of IE. Musculoskeletal symptoms are increasingly reported as prodromal manifestations but are often misattributed to isolated orthopedic or infectious processes. It is highly important to maintain a high suspicion of IE in patients with prosthetic heart vales who present with an unexplained inflammatory or infectious syndromes. Guidelines emphasize the role of multimodal imaging in suspected prosthetic valve IE (transesophageal echocardiography, computed tomography, and positron emission tomography) [9,12].

The presence of negative blood cultures was another diagnostic challenge. In our patient, prior antibiotic exposure for presumed sacroiliitis suppressed bacteremia, leading to negative blood cultures and further diagnostic delay—hallmarks of culture-negative endocarditis, which remains a major obstacle in clinical practice [13,14].

### 4.2. Diagnostic Findings: Mitral Valve Dysfunction and Embolic Phenomena

Concomitant mitral valve involvement was observed, with echocardiographic evidence of severe mitral regurgitation. Infection of the native mitral valve suggests a more aggressive and disseminated disease process. Thus, severe mitral regurgitation worsens hemodynamic stability and significantly increases the risk of heart failure. The presence of both prosthetic aortic and native mitral valve infection underscore the severity of the disease and complicates the management of the case [9,12].

Pulmonary embolism (PE) was also identified in our patient, involving the segmental lingular tributary artery and adding further complexity. Usually thromboembolic events are more frequent in patients with right-sided IE, they may rarely appear in patients with PVE but have been described. The mechanism is likely multifactorial and involves endothelial damage, infection related to hypercoagulability or immobilization. Although PE is uncommon, it contributes to a poor prognosis of a case [15,16,17].

### 4.3. Coronary Complications and Diagnostic Dilemma

A critical complication in this case was acute myocardial infarction caused by septic coronary embolization. Stent placement was performed under the assumption of thrombosis. However, the exact etiology cannot be definitively established. Given the patient’s hemodynamic instability, stenting was considered the most appropriate intervention, with the aim of preserving life despite the inherent risks, but differentiating septic embolism from thrombotic occlusion is extremely difficult during acute coronary syndromes. There are some angiographic clues, such as abrupt vessel cut-off without evidence of underlying atherosclerosis, absence of significant coronary disease on prior angiography, and an irregular, friable appearance of the obstructive material that may suggest septic embolism, especially in patients with concomitant systemic emboli or persistent infection, but angiographic features may overlap, and intravascular imaging is rarely feasible in unstable patients. This distinction, however, is crucial: while percutaneous coronary intervention (PCI) is standard in thrombotic occlusion, stent implantation in septic embolism carries catastrophic risks, including stent infection, mycotic aneurysm, arterial rupture, or persistent sepsis. Our patient deteriorated rapidly after PCI, emphasizing the potential harm of routine revascularization strategies in the setting of active endocarditis. Implanting a stent in a patient with active infective endocarditis carries the risk of secondary stent infection, propagation of septic thrombus and an exaggerated systemic inflammatory response. These mechanisms may have amplified the hemodynamic instability and may have contributed to the rapid progression to refractory shock observed in our patient. Therefore, while PCI was necessary to address the acute coronary occlusion, its potential adverse impact in the setting of uncontrolled infection must be carefully considered. Both STEMI and NSTEMI presentations with angiographic evidence of embolic occlusion are associated with high short-term mortality. Management of acute coronary syndrome in this case is significantly challenging. Current guidelines offer little specific direction for coronary embolism in IE, underscoring the need for further research and clinical consensus [18,19].

### 4.4. Therapeutic Limitations

Fibrinolysis is contraindicated due to the risk of intracranial bleeding from mycotic aneurysm rupture, while PCI carries several significant risks such as stent infections that are potentially lethal, with mortality reported between 40 and 60% in case series [20,21]. Cardiac surgery after TAVI is challenging and carries high operative risk due to advanced age, frailty, and comorbidities of the patient, but it remains the only potentially curative treatment for PVE in the presence of heart failure, persistent or uncontrolled infection, perivalvular abscess formation, and recurrent embolic events. This creates a therapeutic impasse with high mortality, as highlighted in this case. Our patient’s rapid hemodynamic deterioration, cardiogenic shock, and multiple organ dysfunction precluded transfer for surgery. The multidisciplinary endocarditis team considered surgical intervention but judged it infeasible given the acute instability [10,12].

Overall, mortality in patients with PVE after TAVI remains extremely high, especially when it is complicated by myocardial infarction. The incidence of PVE after TAVI is estimated to be between 0.1 and 2.3% and mortality rates for TAVI-associated PVE have been found to extend beyond 30% and surgical reintervention often is limited by comorbidities and frailty. There is a significant proportion of patients that experience persistent infection or embolic events despite aggressive treatment, as previous studies have emphasized [10,11]. In this case, our patient rapidly worsened after coronary angiography and stenting, reflecting a fulminant natural course of TAVI-associated PVE and the added risks of coronary intervention in the presence of active infection. Death occurred from the interaction between multiple factors such as advanced prosthetic-valve infection, myocardial infarction, systemic embolization, and stent implantation under bacterial conditions. This chain illustrates why survival in these situations is limited despite maximal supportive care and why early recognition and individualized treatment strategies are so important to improve the prognosis.

## 5. Conclusions and Future Directions

This case illustrates the multifaceted challenges of diagnosing and managing IE in patients with prosthetic valves after TAVI and underscores the complexity and high mortality rate of TAVI-associated PVE, particularly when the initial presentation is atypical and complicated by prior antibiotic exposure, leading to negative blood cultures. Although the sequence of events in our patient—from musculoskeletal symptoms suggestive of sacroiliitis, to pulmonary embolism, multivalvular infection with severe regurgitation, atrial fibrillation and ultimately coronary embolism leading to STEMI—does not establish novel mechanisms, it underscores several important clinical lessons.

• PVE after TAVI is rare but associated with high morbidity and mortality, requiring a high index of suspicion.
• Atypical manifestations of endocarditis, such as musculoskeletal symptoms (sacroiliitis), can delay recognition and appropriate treatment.
• Negative blood cultures, often due to prior antibiotic exposure, represent a diagnostic challenge in suspected PVE.
• STEMI complicating PVE raises a critical therapeutic dilemma, as distinguishing thrombotic occlusion from septic embolization is essential but often difficult; management requires individualized, multidisciplinary decision-making.

Future studies are needed to improve outcomes in this high-risk population. The research has to establish diagnostic algorithms for early recognition of coronary embolization in IE, to define safe interventional strategies, and to develop consensus on managing this rare but lethal complication of prosthetic valve infection.

Prospective studies are essential in order to expand understanding, to standardize the management of these situations and to improve the survival of patients with TAVI-associated PVE.

## Figures and Tables

**Table 1:** Blood test results on admission.

Test	Result	Reference Range
White Blood Cell Count	10.3 × 10^3^/uI	4.00–10.0 × 10^3^/uI
Lymphocytes	0.94 × 10^3^/uI	1.0–4.0 × 10^3^/uI
Hematocrit	35%	35.0–47.0%
Mean Corpuscular Hemoglobin	26.6 pg	27.9–34.0 pg
Neutrophils	8.85 × 10^3^/uI	2.40–6.50 × 10^3^/uI
Hemoglobin	11 g/dL	12.6–17.4 g/dL
Mean Corpuscular Volume	86.4 fL	80–100 fL
Platelet Count	229 g/dL	150–450 g/dL
Red Blood Cell Count	4.13 × 10^6^/uI	3.80–5.20 × 10^6^/uI
Blood glucose	142 mg/dL	82–115 mg/dL
Aspartate Transaminase	191 U/L	5–34 U/L
Alanine Transaminase	241 U/L	0–55 U/L
Gamma-Glutamyl Transferase	300 U/L	12–64 U/L
Total Bilirubin	1.1 mg/dL	0.2–1.2 mg/dL
Direct Bilirubin	0.59 mg/dL	0.0–0.5 mg/dL
Serum Urea	40 mg/dL	8.4–25.7 mg/dL
Serum Creatinine	1.53 mg/dL	0.72–1.25 mg/dL
C-Reactive Protein	28 mg/L	0–5.0 mg/L

**Table 2:** Blood test results at 48 h after admission.

Test	Result	Reference Range
White Blood Cell Count	8.75 × 10^3^/uI	4.00–10.0 × 10^3^/uI
Lymphocytes	1.08 × 10^3^/uI	1.0–4.0 × 10^3^/uI
Hematocrit	33.9%	35.0–47.0%
Mean Corpuscular Hemoglobin	26.9 pg	27.9–34.0 pg
Neutrophils	6.99 × 10^3^/uI	2.40–6.50 × 10^3^/uI
Hemoglobin	10.9 g/dL	12.6–17.4 g/dL
Mean Corpuscular Volume	83.9 fL	80–100 fL
Platelet Count	205 g/dL	150–450 g/dL
Red Blood Cell Count	4.07 × 10^6^/uI	3.80–5.20 × 10^6^/uI
Blood glucose	150 mg/dL	82–115 mg/dL
Aspartate Transaminase	105 U/L	5–34 U/L
Alanine Transaminase	67 U/L	0–55 U/L
Gamma-Glutamyl Transferase	122 U/L	12–64 U/L
Total Bilirubin	1 mg/dL	0.2–1.2 mg/dL
Direct Bilirubin	0.5 mg/dL	0.0–0.5 mg/dL
Serum Urea	16 mg/dL	8.4–25.7 mg/dL
Serum Creatinine	1.3 mg/dL	0.72–1.25 mg/dL
C-Reactive Protein	45 mg/L	0–5.0 mg/L

## Data Availability

The original contributions presented in this study are included in the article. Further inquiries can be directed to the corresponding authors.
